# Signaling Mechanism of Budding, Proliferation, and Tissue Regeneration in Cnidaria

**DOI:** 10.3390/cimb47040219

**Published:** 2025-03-24

**Authors:** Jie Lv, Jinhong Chen, Liangzhi Li, Xiaoyu Geng, Bingbing Li, Mingke Wang, Jishun Yang

**Affiliations:** 1 Health Science and Engineering, University of Shanghai for Science and Technology, Shanghai 200093, China; lj0419@smmu.edu.cn; 2 Naval Medical Center of PLA, Naval Medical University, Shanghai 200052, China; 15618421309@163.com (J.C.); gxy0827@smmu.edu.com (X.G.); m18131452435@163.com (B.L.); 3 College of Marine Sciences, Shanghai Ocean University, Shanghai 201306, China; 1431390050@163.com

**Keywords:** cnidaria, budding, tissue regeneration, development, signaling pathways, stem cells

## Abstract

Phylum Cnidaria occupies an early branching position in the evolution of eukaryotes, establishing both close and distant relationships with most other eukaryotic metazoans. Budding encompasses the complete processes of cell proliferation, differentiation, and tissue regeneration, making it an ideal model for exploring various aspects of cellular function and evolution. Additionally, budding serves as the primary reproductive method for increasing the cnidarian population. This asexual reproductive phase is critical for managing and mitigating cnidarian outbreaks. This paper summarizes the common factors influencing budding, the signaling pathways involved and their associated functions, and the methodologies employed in relevant research, providing a theoretical foundation for the prevention and control of cnidarian populations.

## 1. Introduction

### 1.1. Cnidarian Life Cycle Overview

Cnidarians are radially or biradially symmetrical metazoans with two embryonic layers, named for their specialized stinging cells called *Cidocytes*. The phylum comprises three orders: hydrozoa (e.g., *hydras*), anthozoa (e.g., *corals*), and scyphozoa (e.g., *jellyfish*). These organisms exhibit a polyp stage in their life cycle, which facilitates asexual reproduction through budding. Species such as *Aurelia aurita* (*moon jellyfish*) and *coral polyps* demonstrate variations in polyp retention, with corals forming colonies through persistent attachment of buds to the parent body [1,2].

### 1.2. Budding Mechanisms in Hydrozoans

**Figure 1:**
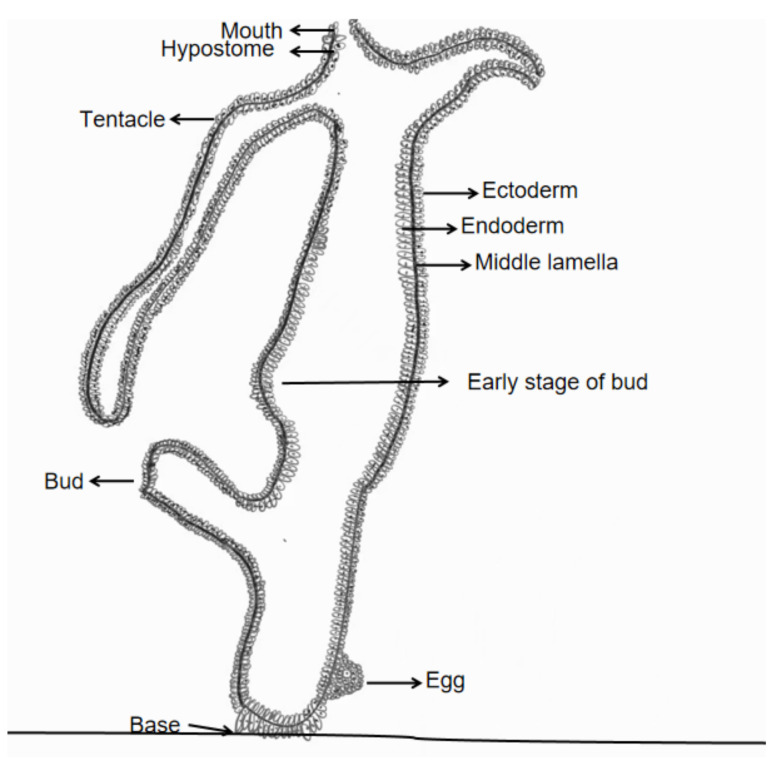
Vertical section of a Hydra.

Budding in cnidarians involves localized cell proliferation and differentiation to form new individuals. In hydras, budding initiates with an outward protrusion of the body wall, which develops into a bud connected to the parent via shared digestive and circulatory cavities. Key structures like the hypostome (a conical region surrounding the mouth) and tentacles differentiate within the bud before detachment via basal disk contraction [3,4]. Unlike hydras, coral polyps retain buds to build colonial structures. Hydrozoans exhibit remarkable adaptability, sustaining asexual reproduction even under suboptimal environmental conditions to maintain population stability. Quantitative models of polyp population dynamics, such as exponential growth equations (*N_t_* = *N*_0_*e^rt^*) or logistic frameworks incorporating carrying capacity (*dN*/*dt* = *rN*(1 − *N*/*K*), highlight the potential for rapid outbreak formation when budding rates exceed natural mortality [5,6,7,8,9]. The vertical section of hydra is shown in Figure 1.

### 1.3. Species-Specific Variations in Budding

Asexual reproduction strategies differ significantly across cnidarian taxa. While hydras release independent buds, jellyfish polyps (e.g., *Aurelia*) retain ancestral genetic programs critical for metamorphosis into medusa stages [10,11,12]. Some Hydrozoa and Anthozoa species have lost genes essential for medusa formation, yet these genes remain active during jellyfish polyp stages, underscoring their evolutionary significance [13]. These variations highlight the phylum’s diversification and adaptive plasticity.

### 1.4. Genomic and Evolutionary Insights

Cnidarians provide a unique window into early metazoan evolution due to conserved genetic pathways shared with vertebrates. Their diploblastic body plan (two embryonic layers) represents a precursor to the triploblastic structure of bilaterally symmetrical animals [9]. Budding involves stem cell proliferation regulated by pathways homologous to those in mammalian systems, such as Wnt and Notch. Studies of cnidarian stem cells—particularly in polyps, which possess three distinct lineages—offer high-resolution models for investigating tissue regeneration and differentiation [14,15].

### 1.5. Ecological Impacts of Cnidarian Proliferation

Jellyfish blooms, driven by polyp-stage budding, pose significant ecological and economic challenges. Species like *Nemopilema nomurai* and *Aurelia coerulea* disrupt fisheries, power plant operations, and tourism through mass aggregations [16,17,18]. Their predation on fish eggs and competition with planktonic fish alter marine food webs, often correlating with declining fish populations [17,18]. Computational approaches, such as individual-based models (IBMs) simulating polyp colony growth under varying temperatures or nutrient conditions, predict critical thresholds for bloom initiation. Effective management requires understanding the polyp stage’s reproductive potential, yet research remains limited compared to freshwater hydra models [5,19].

### 1.6. Signaling Pathways in Budding and Regeneration

Signaling pathways coordinate cell behavior during budding. Key pathways include: Wnt/β-catenin (regulates axial patterning and stem cell fate), Hippo (controls organ size via cell proliferation inhibition), PI3K and JNK (mediates stress responses and morphogenesis), and FGFR and Notch (direct cell differentiation and tissue boundaries) [10,20,21,22]. These pathways are conserved across metazoans, with hydra regeneration studies providing insights into their roles in tissue repair and asexual reproduction. Biomechanical models, incorporating tissue stiffness and hydrostatic pressure gradients, further elucidate how physical forces interact with these molecular pathways to shape bud morphogenesis.

### 1.7. Hydra as a Model Organism

The freshwater hydra’s structural simplicity, transparency, and regenerative capacity make it a cornerstone of developmental biology. Its diploblastic body comprises ectoderm, endoderm, and a gelatinous mesoglea (extracellular matrix). Cell types include epithelial muscle cells, nerve cells, and pluripotent interstitial stem cells. Budding and regeneration studies often involve surgical excision of tissue to dissect signaling pathway dynamics, revealing mechanisms applicable to broader metazoan biology. Recent computational frameworks, such as reaction-diffusion models and cellular automata, simulate the self-organization of hydra tissues during budding, offering predictive tools for manipulating asexual reproduction rates [23,24,25,26].

Despite their significance as model organisms, several critical gaps remain in our understanding of cnidarian biology. First, while the molecular mechanisms underlying hydra budding have been extensively studied, much less is known about budding regulation in marine cnidarians, particularly jellyfish polyps. Second, the ecological significance of budding in mediating population dynamics and outbreak formation remains poorly understood. Integrative studies combining genomic data with spatially explicit population models could bridge this gap, enabling predictions of bloom scenarios under climate change. Third, comparative studies of budding mechanisms across different cnidarian taxa are scarce, limiting our ability to draw general conclusions about the evolution of asexual reproduction in this phylum. This review aims to synthesize current knowledge on the cellular and molecular mechanisms of cnidarian budding, with particular emphasis on conserved signaling pathways, quantitative models of outbreak dynamics, and computational approaches to budding regulation. By integrating molecular, developmental, and ecological perspectives, we hope to provide a comprehensive understanding of cnidarian budding and its broader biological significance.

## 2. Common Methods for Studying Budding and Tissue Regeneration in Cnidaria

Decades of scientific research have led to the development of established methodologies for studying budding, proliferation, and tissue regeneration in cnidarians. Common techniques include in situ hybridization, fluorescence in vivo tracing, signaling pathway inhibitors, gene knockout, and gene overexpression. Research by Meinhardt et al. provided a theoretical framework elucidating the mechanisms underlying phenomena observed in these methodologies, particularly how organisms can form structural sequences capable of intercalary or terminal regeneration through relatively simple molecular interactions [23]. The rapid advancement of single-cell transcriptome sequencing technology has also been fully integrated into this field, enhancing our understanding of signaling pathways related to the proliferation of specialized cells.

In situ hybridization involves the hybridization of nucleic acid probes labeled with specific markers to target nucleic acids in cell or tissue sections, allowing for accurate and quantitative detection of specific sequences. While this method provides spatial resolution, its quantitative accuracy depends critically on probe specificity and hybridization efficiency, with potential cross-reactivity artifacts requiring rigorous negative controls. In hydras, gene probes are employed to introduce key genes and induce the expression of related proteins and signaling pathways, thereby providing insights into the roles of these pathways in bud proliferation [27]. Fluorescence in vivo tracing is utilized in hydras to visualize the proliferation, migration, and differentiation of various cell types within these transparent cnidarians. While offering high spatiotemporal resolution, this method faces limitations in accuracy due to phototoxicity-induced cellular stress (particularly during long-term imaging) and signal attenuation in deep tissue layers (>100 μm), potentially skewing proliferation rate measurements. Reproducibility is further challenged by variability in fluorescent marker expression efficiency across specimens. Comparative studies show fluorescence tracing achieves superior temporal resolution but lower quantitative accuracy than endpoint methods like in situ hybridization [28,29]. Signaling pathway inhibitors are commonly used to investigate the relationship between specific signaling pathways and cnidarian proliferation. Notable inhibitors include: Apigenin (an ERK signaling pathway inhibitor), LY294002 (a PI3K signaling pathway inhibitor), and SU5402 and SU5416 (inhibitors of VEGF/FGF signaling pathways). While inhibitor studies allow rapid pathway interrogation, their accuracy is compromised by off-target effects. For instance, LY294002 exhibits known cross-reactivity with DNA-PK and mTOR at common concentrations. Reproducibility varies significantly between inhibitor classes, with small-molecule inhibitors like SU5402 showing batch-dependent potency variations of up to 40% [30]. These limitations necessitate complementary approaches like genetic knockout for conclusive pathway validation. Gene knockout and overexpression techniques are also employed to explore budding, proliferation, and tissue regeneration in cnidarians. In Hydra models, functional redundancy across paralogous genes frequently results in attenuated phenotypic changes, reducing both the apparent effect size and experimental reproducibility. Knockout efficiency varies substantially between target genes (30–85% success rates reported), with incomplete penetrance potentially masking true biological effects. While CRISPR-based methods have improved accuracy over earlier RNAi approaches, off-target editing rates of 5–15% still complicate interpretation [31]. Furthermore, single-cell RNA sequencing technology significantly contributes to the study of cell differentiation, phenotyping, subtype classification, and pseudo-temporal analysis of cell differentiation. Despite its high molecular resolution, this method suffers from technical variability in cell capture efficiency (typically 10–50% per channel) and transcript detection sensitivity, potentially biasing cell type abundance measurements. While demonstrating excellent reproducibility in cell clustering between technical replicates (median correlation > 0.95), spatial information loss limits direct comparison with imaging-based methods [32].

Current methods face additional technical and biological constraints. Fluorescence tracing remains limited by phototoxicity and imaging depth in thick tissues [28]. Gene knockout in species with high genetic redundancy (e.g., Hydra) often yields subtle phenotypes, complicating functional studies [31]. Single-cell RNA sequencing requires high sample quality, with even minor RNA degradation (>10% DV200) significantly altering cluster resolution [32]. Inhibitors like LY294002 (PI3K) may have off-target effects, obscuring pathway-specific roles [30]. Method selection involves critical trade-offs: while fluorescence tracing and single-cell sequencing offer dynamic and comprehensive profiling respectively, they lack the causal resolution of genetic manipulation. Conversely, inhibitor studies and knockout models provide pathway-specific insights but require validation through orthogonal methods due to their respective limitations in specificity and penetrance [33].

The common methodologies for investigating budding proliferation and tissue regeneration in cnidarians are summarized in Table 1.

## 3. Influence Factors on the Budding and Proliferation

The budding and proliferation of cnidarians are regulated by a suite of environmental and biological factors. Under standard conditions, food availability, temperature, and salinity play dominant roles, while pH, symbiotic relationships, and pollutants become significant under extreme or altered environments. Emerging systems biology perspectives reveal that these factors collectively modulate a core regulatory axis involving β-catenin, YAP/TAZ, and MAPK signaling, which dynamically balances cell proliferation with stress responses.

### 3.1. Feeding Level

Hydras maintain a starvation-resistant proliferative state through metabolic plasticity. Optimal feeding sustains Wnt/β-catenin-driven cell production, with excess cells migrating to budding sites. The system exhibits bistability: starvation suppresses β-catenin activity and upregulates autophagy, while overfeeding paradoxically reduces efficiency through ROS accumulation and MAPK pathway inhibition. This dual regulation prevents uncontrolled proliferation under fluctuating nutrient conditions [34,35].

### 3.2. Temperature

Thermal effects on budding demonstrate pathway cross-talk: in *Aurelia coerulea*, 15 °C optimally activates β-catenin-mediated proliferation while suppressing Hippo/YAP-mediated stress signaling. Extreme temperatures induce pathway switching—heat shock activates p38 MAPK to arrest cell cycle progression, while cold stress upregulates Hippo signaling to promote dormancy. These temperature-sensitive thresholds create a “Goldilocks zone” for budding through competing pathway activities [7,36].

### 3.3. Salinity

Salinity shifts trigger a systems-level response where osmosensing pathways (PI3K/ERK) interact with the core proliferation-stress axis. Optimal salinity maintains ERK-mediated β-catenin stabilization. Hyposalinity activates Hippo/YAP to redirect resources to podocyst formation, while hypersalinity induces oxidative stress that simultaneously inhibits Wnt and MAPK pathways. This salt-dependent signaling hierarchy prioritizes survival over reproduction [37,38].

### 3.4. Other Factors

Freshwater hydras maintain pH homeostasis through MAPK-regulated ion transporters [33,39], whereas marine species exhibit greater pH tolerance through symbiotic alkalization [40,41,42,43]. Pollutants disrupt network balance through multiple mechanisms: heavy metals (Cd, La, Co, Cu, Zn) preferentially inhibit β-catenin nuclear translocation [44], whereas microplastics impair ERK signaling through physical membrane interactions [45].

Environmental factors such as temperature, salinity, and food availability modulate budding by influencing key signaling pathways. For instance, optimal temperatures (e.g., 15 °C for *Aurelia coerulea*) enhance metabolic efficiency, activating Wnt/β-catenin and MAPK pathways to promote cell proliferation and differentiation [36,46]. Conversely, salinity fluctuations may suppress budding via stress-induced Hippo pathway activation, which inhibits YAP/TAZ-mediated growth [37,47]. Genetic factors, including conserved regulators like β-catenin and YAP, integrate environmental signals to orchestrate tissue patterning and bud formation [48,49]. Synergistically, environmental stressors such as hypoxia or pollutants (e.g., microplastics) disrupt metabolic pathways (e.g., PI3K/ERK), indirectly altering gene expression linked to stem cell maintenance [44,50]. This interplay highlights the dynamic balance between external conditions and intrinsic genetic networks in regulating budding efficiency.

The common factors affecting Hydra budding are summarized in Table 2.

## 4. Cell Types and Their Roles Involved in Budding and Proliferation of Prickly Cell Animals

Cnidarian growth, budding, and tissue regeneration rely on the proliferation and differentiation of stem cells. Two primary stem cell types drive these processes: epithelial muscle cells (mitotic mononuclear cells in the body column) and mesenchymal stem cells (pluripotent cells in the ectoderm/endoderm). Mesenchymal stem cells differentiate into specialized cell types—cnidoblasts, neurons, glandular cells, and germ cells—while epithelial cells self-renew and migrate to form structural tissues [19,51].

### 4.1. Epithelial Muscle Cells

Epithelial cells in hydras are categorized into ectodermal and endodermal subtypes. Ectodermal cells migrate toward extremities, differentiating into tentacle, hypostome, or basal disk cells, while endodermal cells form gastric layers and mucous gland cells. Both subtypes exhibit high self-renewal capacity and maintain polarity through interactions with surface microbiota [52,53,54,55,56,57].

### 4.2. Cnidoblasts

Derived from mesenchymal stem cells, cnidoblasts house nematocysts for prey capture and defense. These stinging structures discharge via sialic acid-mediated triggers, a mechanism mimicked experimentally using nanoparticles. Post-differentiation, cnidoblasts migrate to tentacles or body surfaces, with their biomechanical discharge processes inspiring bionic research [58,59,60,61].

### 4.3. Neural Cells

Neural differentiation in hydras occurs in ectodermal regions (head, foot) under neuropeptide regulation. Activator-inhibitor concentration gradients determine whether mesenchymal stem cells become neurons or cnidoblasts. Neurons contribute to budding, regeneration, and microbiota modulation via antimicrobial neuropeptides [62,63,64,65].

### 4.4. Germ Cells

Germ cells arise from mesenchymal stem cells, forming gonads for sexual reproduction. Under stress, hydras prioritize germ cell differentiation to ensure reproductive success. Hermaphroditic species exhibit gender plasticity, with germ cell dynamics trackable via fluorescence imaging [29,55,56,61].

### 4.5. Glandular Cells

Glandular cells in tentacles, basal disks, and gastric layers secrete enzymes, glycoproteins, and toxins. In skeletal cnidarians (e.g., corals), they produce structural materials like chitin or calcium. These cells also express toxin genes, linking secretory functions to ecological interactions [63,64].

While Hydra’s simplicity and regenerative capacity make it a valuable model, its applicability to marine cnidarians is limited. Hydra lacks a medusa stage, limiting insights into jellyfish life cycles [12]. Its freshwater habitat also contrasts with marine species’ salinity-dependent budding mechanisms [37]. Additionally, Hydra’s stem cell lineages differ from corals, which rely on symbiotic algae for proliferation [40,43]. Genetic redundancy in Hydra may obscure phenotypic effects of gene knockouts, unlike in species with lower regenerative capacity [31]. Thus, findings in Hydra require validation in marine cnidarians to ensure broader relevance.

Cell types and functions involved in the budding and proliferation of cnidarians are shown in Table 3.

## 5. Signaling Pathways and Their Effects on the Proliferation and Sprouting of Prickly Cell Animals

The pathways (e.g., Wnt, Hippo, MAPK) were selected based on their conserved roles in metazoan development and extensive evidence in cnidarian studies. Wnt/β-catenin is central to axial patterning in Hydra and corals, while Hippo regulates tissue homeostasis across species [47,65]. MAPK and ERK pathways were included due to their involvement in stress responses and regeneration, which are critical for budding under variable conditions [46,66]. Pathways like Notch and BMP were prioritized for their evolutionary significance in boundary formation and cross-talk with other signals [67,68]. This selection reflects pathways with both functional importance and homology to vertebrate systems, enabling comparative evolutionary analyses.

### 5.1. Wnt/β-Catenin Signaling Pathway

The Wnt/β-catenin pathway governs axial patterning and regeneration in hydras. β-catenin is essential for head/foot regeneration, with its nuclear localization driving tissue organizer formation, while its loss blocks regeneration [48,69]. AXIN1, in complex with APC/GSK3β/CK1α, regulates β-catenin degradation [70]. Wnt signaling also interacts with other pathways (e.g., Hippo, BMP) to establish body axes, reflecting its conserved role in coordinating polarity and regeneration [55,71].

### 5.2. Hippo Signaling Pathway

Hippo signaling maintains tissue homeostasis via YAP, a key effector enriched in proliferative zones (e.g., digestive tract, budding regions) [49,72]. YAP inhibition accelerates budding, while its interaction with 14-3-3 and LATS fine-tunes growth [73,74]. Notably, Hippo regulates Wnt upstream, linking cell proliferation to somatic axis formation—a mechanism conserved in bilaterians [47,55].

### 5.3. Mitogen-Activated Protein Kinase (MAPK) Signaling Pathway

The MAPK family (ERK, JNK, p38) regulates proliferation, stress responses, and regeneration. ERK drives budding and head organizer formation, while JNK supports compensatory proliferation post-injury [75]. Src kinases, activated by MAPK, mediate foot development [46]. STK, a Src homolog, further underscores MAPK’s role in polyp differentiation [76]. These functions are conserved in bilaterally symmetrical animals [26], paralleling mechanisms in jellyfish collagen matrices that maintain chondrocyte phenotypes via integrin-like interactions [77].

### 5.4. PI3K Signaling Pathway

PI3K refers to a family of kinases involved in crucial cellular processes such as growth, proliferation, differentiation, motility, survival, and intracellular transport. The PI3K signaling pathway is implicated in the early stages of head development in polyps, particularly during the onset of cell differentiation and the formation of head tissue conductors. PI3K inhibitors block early budding but not late-stage development, while LY294002 suppresses head regeneration via PKB downregulation [22,46,50]. This pathway’s stage-specific roles highlight its regulatory precision.

### 5.5. JNK Signaling Pathway

C-Jun amino-terminal kinase (JNK), a subclass of MAPKs, is crucial in various physiological and pathological processes, including the cell cycle, reproduction, apoptosis, and cellular stress responses. JNK, a MAPK subclass, is selectively activated during spine differentiation and apoptosis-driven compensatory proliferation in hydras [44,71]. Its limited role in budding underscores functional specialization within the MAPK network.

### 5.6. ERK Signaling Pathway

ERK, another member of the MAPK family, is central to the signaling network that regulates cell growth, development, and division. ERK is central to budding and head organizer development. Apigenin-mediated ERK inhibition blocks budding and tissue conductor formation [46,66]. FGFR-MEK/ERK crosstalk facilitates tissue separation during budding, while ERK’s role in wound healing is conserved in Nematostella [78,79].

### 5.7. Protein Kinase C (PKC) Signaling Pathway

The PKC family of proteins plays a significant role in various cellular functions, including cell proliferation, apoptosis, gene transcription and translation, cellular morphology, and intercellular contact regulation. PKC regulates proliferation and differentiation during head regeneration but has minimal impact on budding [46]. Its multifunctional roles (e.g., enzyme activation, gene regulation) highlight context-dependent signaling.

### 5.8. FGFR Signaling Pathway

Fibroblast growth factors (FGFs) bind to their respective receptors (FGFRs) to activate downstream signaling pathways that are vital for both proliferative processes (such as embryogenesis, growth, and development) and non-proliferative processes (including neuroregulation and metabolic control). FGFR activates MEK/ERK to drive tissue separation during budding [78,80]. Its dual role in proliferative (embryogenesis) and non-proliferative (neuroregulation) processes underscores evolutionary versatility.

### 5.9. Bone Morphogenetic Protein (BMP) Signaling Pathway

BMP signaling, a TGF-β superfamily member, antagonizes Wnt via Dickkopf induction in glandular cells, balancing organizer activity [67]. Hysmad, a BMP homolog, regulates tentacle formation and regeneration at their bases [8]. This pathway’s dual role in suppressing Wnt and patterning tentacles suggests ancient origins in axial coordination, mirroring vertebrate mesoderm regulation. Further studies are needed to explore BMP-Notch crosstalk in boundary formation.

### 5.10. Notch Signaling Pathway

Notch establishes parent-bud boundaries through HyHes and matrix metalloproteinase A3 [68,81]. HyJagged defines the separation interface, while Notch1-CSL interactions convert transcriptional repression to activation, suppressing Noggin [27,82,83]. This pathway’s role in segregating germline and somatic fields highlights its early evolutionary origin in spatial patterning.

### 5.11. Vascular Endothelial Growth Factor (VEGF)/FGF Signaling Pathway

VEGF/FGF synergism enables hypostome and tentacle regeneration. Inhibitor studies show delayed head regeneration and reduced expression of head-specific genes (HyBra1, HyKs1, HyAlx) [84], underscoring their role in tissue morphogenesis. Interactions between signaling pathways involved in the budding and proliferation of cnidarians include Hippo regulation of Wnt to coordinate growth and polarity [47], BMP-induced Dickkopf antagonism of Wnt to balance organizer activity [67], and Notch boundaries guiding MAPK-driven proliferation zones [68]. These converge on head organizer development [46,66].

Current knowledge of pathway cross-talk is fragmented. For example, while Hippo-Wnt interactions are documented in Hydra, their conservation in marine species remains unverified [47]. BMP-Wnt antagonism in glandular cells is inferred from Dickkopf expression but lacks direct mechanistic evidence [72]. Most studies focus on pairwise interactions, neglecting broader network dynamics. Technical limitations, such as the absence of dual pathway inhibitors or multi-omics integration, hinder comprehensive analyses. Resolving these gaps requires transgenic models and spatial transcriptomics to map pathway interactions in real-time. An overview of the signaling pathways and their associated functions involved in the budding and proliferation of cnidarians is presented in Table 4.

**Figure 2:**
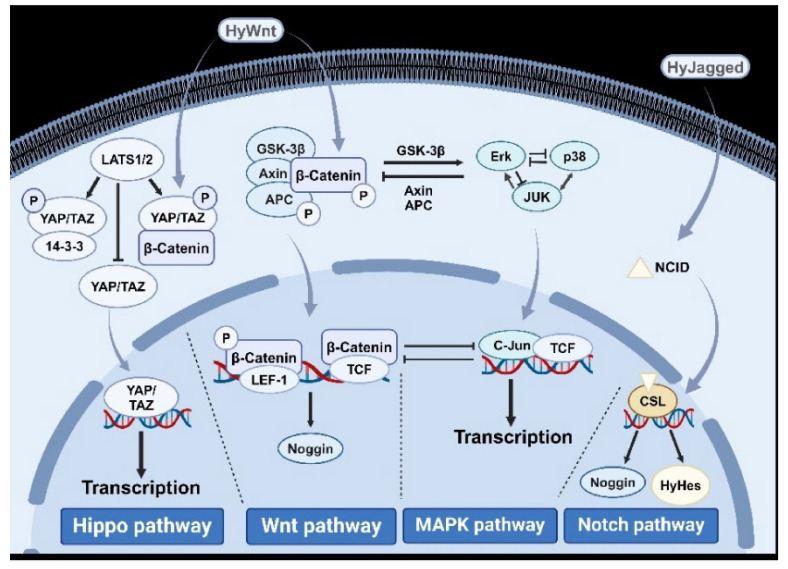
Signaling pathway map involved in the budding and proliferation of cnidarians.

Based on the above introduction, the most common signaling pathways that affect bud proliferation in cnidarians include the Hippo, Wnt, MAPK, and Notch pathways. We have illustrated a signal pathway diagram, as shown in Figure 2.

Based on the preceding discussion, the most prevalent signaling pathways influencing bud proliferation in cnidarians include the Hippo, Wnt, MAPK, and Notch pathways. We have illustrated these pathways in a diagram, as shown in Figure 2.

## 6. Outlook

Cnidarians, as diploblasts, represent some of the simplest animals at the tissue level. Their developmental plasticity enables processes such as metamorphosis, regeneration, and asexual reproduction, making them pivotal for studying evolutionary and developmental biology. The polyp stage, crucial for population expansion, is influenced by external factors (temperature, salinity, feeding) and intrinsic signaling pathways. At a cellular level, budding involves proliferation, migration, and differentiation of stem and somatic cells, coordinated by pathways like Wnt, Hippo, MAPK, and BMP. These pathways respond to extracellular stimuli, producing effector proteins that modulate budding [36,39,45]. Below, we synthesize these mechanisms and introduce their potential applications across diverse fields.

Cnidarian signaling pathways offer profound insights for regenerative medicine and disease control. The conserved Wnt/β-catenin pathway, which governs stem cell dynamics and tissue polarity in Hydra [85,86], shares homology with human pathways regulating tissue repair and cancer progression. Targeting Wnt-Hippo cross-talk, which balances proliferation and differentiation in cnidarians, could inspire therapies for degenerative diseases or tumor suppression [87]. For instance, Hippo’s inhibition of Wnt in Hydra [47] mirrors pathways controlling organ size in mammals, suggesting strategies to modulate tissue overgrowth in cancers. Additionally, Hydra’s rapid cell turnover provides a model for studying anti-aging mechanisms or optimizing in vitro stem cell cultures for transplantation [26]. Epigenetic regulation in cnidarians, such as nutrient-dependent chromatin remodeling during tentacle regeneration [84], may further elucidate environmental impacts on human stem cell differentiation, offering routes to enhance wound healing or engineered tissue maturation.

Cnidarians inspire bioinspired materials design. Coral polyps, which biomineralize calcium carbonate skeletons under pH stress [39], could guide the development of self-assembling, pH-responsive materials for bone regeneration or marine construction. Jellyfish mesoglea, a resilient yet flexible extracellular matrix, presents a template for synthetic hydrogels with applications in soft robotics or drug delivery systems. Furthermore, the self-organizing properties of cnidarian tissues during budding—regulated by Notch and MAPK boundaries [68]—could inform adaptive materials capable of autonomous repair or structural reconfiguration in response to damage, akin to “living” building materials.

The complexity of cnidarian signaling networks and their environmental sensitivity positions them as ideal systems for AI-based simulations. Machine learning algorithms could model how ocean warming alters Hippo-Wnt cross-talk to predict polyp population declines [36,45], aiding conservation strategies. Computational frameworks might simulate pathway synergies (e.g., BMP-Dkk-Wnt [67]) to identify therapeutic targets for human diseases linked to dysregulated growth, such as fibrosis or cancer. AI could also optimize synthetic biology workflows—for example, predicting how epigenetic modifiers [43,84] enhance Hydra’s regeneration to improve CRISPR-based tissue engineering. Lastly, digital twin models of coral colonies, integrating genomic and environmental data, might forecast bleaching impacts and guide reef restoration efforts.

Climate change disrupts cnidarian budding through ocean warming, acidification, and pollution, destabilizing colonies and reducing asexual reproduction [16,36,39]. These stressors synergize with anthropogenic pollutants to inhibit conserved pathways (e.g., Wnt, Hippo), threatening biodiversity [45]. However, species-specific adaptations exist: Hydra’s stress-resistant asexual dominance contrasts with coral reliance on symbionts [40], while jellyfish polyps exhibit unique metamorphic plasticity [12]. Such diversity highlights both conserved principles and ecological constraints, emphasizing the need for tailored conservation approaches.

Interdisciplinary studies merging cnidarian biology with biomedicine, materials science, and AI could yield transformative breakthroughs. Prioritizing cross-talk between Wnt-Hippo pathways may unveil universal mechanisms of tissue polarity, applicable to organoid engineering. Concurrently, exploring cnidarian biomaterials and epigenetic resilience could drive innovations in sustainable materials and climate adaptation strategies. Finally, AI models bridging molecular networks with ecosystem dynamics will be critical for mitigating anthropogenic impacts on marine ecosystems while advancing human health and technology.

## Figures and Tables

**Table 1:** Common methods for studying budding proliferation and tissue regeneration in cnidarians.

Number	Technology	Function	Advantage	Disadvantage	Author	Ref.
1	In situ hybridization	A gene probe was introduced into Hydra to locate the expression results and expression amount of key genes and related proteins, gaining insight into the role of signal pathways in bud proliferation.	The spatial distribution of RNA in tissues or cells is directly mapped to retain morphological information. No need to rely on genetically modified animals, suitable for non-model organisms. The cost is relatively low, and the technology is mature.	Sensitivity is limited, and low abundance RNAs may not be detectable. The steps (fixation, slicing, probe design) are complicated and take a long time. It is not possible to dynamically observe real-time changes in gene expression.	Perween et al.	[33]
2	Fluorescence tracing in vivo	Introduction of fluorescent protein in vivo and imaging tracing	Real-time dynamic observation of cell migration, differentiation, or molecular activity (such as calcium signaling). It is suitable for long-term tracking of live samples to reduce the interference of sample processing. High specificity can be achieved in combination with transgenic fluorescent labeling.	Depending on the fluorescent labeling technology, phototoxicity or photobleaching effects may be introduced. Imaging depth is limited and is not suitable for thick tissue or large samples. Expensive equipment (such as confocal microscopes) and complex data analysis are required.	Böttger et al.; Khalturin et al.	[28,29]
3	Signal pathway inhibitor	Specific inhibition of signal pathway to regulate bud proliferation	Rapid and reversible blocking of specific pathways to study short-term effects. Simple operation (such as drug immersion or local injection), and low cost. It is suitable for screening key regulatory pathways.	There may be off-target effects that interfere with other pathways. Inhibitor concentration and duration of action need to be strictly optimized; otherwise, false positives/negatives may occur. It is not possible to distinguish the specific functions of different components in the same pathway.	Berking et al.	[30]
4	Knockdown/ Overexpression	Inactivate or overexpress the target gene	Stable genetic strains can be constructed to support long-term research. Rapid verification of gene function gain effect, suitable for phenotypic screening. The spatiotemporal specificity of expression can be controlled by inducible promoters.	Genetic redundancy may result in phenotypic insensitivity (especially in species with high regenerative capacity). The construction time is long (such as stable mutant screening), and the cost is high. Overexpression may exceed physiological levels, leading to artificial illusions.	Beermann et al.	[31]
5	Single Nuclei RNA Sequencing snRNA-seq	Comments on cell differentiation, signal pathways and key molecules	Cell heterogeneity is resolved to identify rare cell types or states. Comprehensive mapping of gene expression to reveal new regulatory factors or pathways. Data can be integrated into multiple omics analyses (e.g., spatial transcriptome).	High cost (sample preparation, sequencing, computing resources). Loss of spatial position information (combined with in situ hybridization verification). Sensitive to sample quality (high requirements for cell activity and RNA integrity).	Camara et al.	[32]

**Table 2:** Common and multiplication factors affecting Hydra budding.

Number	Impact Factor	Function	Author	Ref.
1	Food	Less food supply promotes asexual proliferation	Purcell et al.; Shostak et al.	[34,35]
2	Temperature	15 °C is the optimal temperature for sprouting and proliferation	Jianbin et al.	[7]
3	Salinity	20–32‰ is the optimal salinity for sprouting and proliferation	Xing et al.	[37]
4	O_2_	Low/extremely low oxygen reduces the germination efficiency	Fu et al.; Ishii et al.	[18,37]
5	PH	The optimal pH is 7	Perween et al.; Chuard et al.	[33,38]
6	Microplastics	Obstruct the sprouting and proliferation of polyps	Eom et al.	[44]
7	Heavy metal	Lower the sprouting and proliferation	Zhang et al.	[45]

**Table 3:** Cell types and functions involved in the budding and proliferation of cnidarians.

Number	Cell Type	Function	Author	Ref.
1	Epithelial muscle cell	The types of gastric epidermal cells proliferate to promote the digestive function of polyps and promote budding.	Siebert et al.; Bosch et al.; Vogg et al.	[52,53,54]
2	Prickle cell	Prey, attack, defense	Babonis et al.; Bode et al.	[58,60]
3	Nerve cell	At various stages of budding, proliferation and tissue regeneration of Hydra	Wittlieb, J.; Columbus-Shenkar, Y.Y.	[62,63]
4	Germ cell	Ensure the occurrence of sexual reproduction	Unni, M.; He, J.;Sebestyén et al.;	[55,56,61]
5	Glandular cell	Help polyps attach, prey, and digest	Columbus-Shenkar et al.; Yap et al.	[63,64]

**Table 4:** Signaling pathways and functions involved in the budding and proliferation of cnidarians.

Number	Impact Factor	Regulation	Author	Ref.
1	Wnt	Coding polyp mouth-exit axis body	Lengfeld et al.; Wang et al.	[21,48]
2	β-catenin	Promote the regeneration of head and feet	Gufler et al.	[69]
3	Hippo	Regulation and induction of the formation of new body axis of bud	Brooun et al.	[47]
4	MAPK	Promote head and foot regeneration and bud proliferation	Fabila et al.; Cardenas et al.	[46,85]
5	PI3K	Promote the growth of the head and bud of polyps	Fabila et al.	[46]
6	JNK	Regulating the differentiation and proliferation of prickle cells; regulating compensatory proliferation	Chera et al.; Philipp et al.	[26,76]
7	ERK	Promote polyp sprouting and regulate head development	Sewing, J.; Hasse, C.	[78,79]
8	PKC	Promote head regeneration	Fabila et al.	[46]
9	FGFR	Promote the separation of polyps and buds	DuBuc, T.Q.; Holz, O	[80,81]
10	BMP	Participate in the growth of columnar gland cells	Brooun et al.	[67]
11	Notch	Promote bud differentiation	Iommelli, F.	[83]
12	VEGF/FGF	Promote the regeneration of the head and tentacles	Hu, Y.	[84]
